# Lung Ultrasound Score in Neonates with Congenital Diaphragmatic Hernia (CDH-LUS): A Cross-Sectional Study

**DOI:** 10.3390/diagnostics13050898

**Published:** 2023-02-27

**Authors:** Chiara Maddaloni, Domenico Umberto De Rose, Sara Ronci, Iliana Bersani, Ludovica Martini, Stefano Caoci, Irma Capolupo, Andrea Conforti, Pietro Bagolan, Andrea Dotta, Flaminia Calzolari

**Affiliations:** 1 Neonatal Intensive Care Unit, “Bambino Gesù” Children’s Hospital IRCCS, 00165 Rome, Italy; 2 Neonatal Surgery Unit, “Bambino Gesù” Children’s Hospital IRCCS, 00165 Rome, Italy; 3 Department of Systems Medicine, University of Rome “Tor Vergata”, 00133 Rome, Italy

**Keywords:** LUS, newborns, ultrasonography, congenital malformation

## Abstract

***Introduction:*** The use of a lung ultrasound (LUS) score has been described in the early phases of neonatal respiratory distress syndrome; however, there is still no data regarding the application of the LUS score to neonates with a congenital diaphragmatic hernia (CDH). The objective of this observational cross-sectional study was to explore, for the first time, the postnatal changes in LUS score patterns in neonates with CDH, with the creation of a new specific CDH-LUS score. ***Methods:*** We included all consecutive neonates with a prenatal diagnosis of CDH admitted to our Neonatal Intensive Care Unit (NICU) from June 2022 to December 2022 who underwent lung ultrasonography. Lung ultrasonography (LUS) was determined at scheduled time points: (T0) during the first 24 h of life; (T1) at 24–48 h of life; (T2) within 12 h of surgical repair; (T3) a week after the surgical repair. We used a modified LUS score (CDH-LUS), starting from the original 0–3 score. We assigned 4 as a score in the presence of herniated viscera in the hemithorax (liver, small bowel, stomach, or heart in the case of a mediastinal shift) in the preoperative scans or pleural effusions in the postoperative scans. ***Results:*** We included in this observational cross-sectional study 13 infants: twelve/13 had a left-sided hernia (2 severe, 3 moderate, and 7 mild cases), while one patient had a right-sided severe hernia. The median CDH-LUS score was 22 (IQR 16–28) during the first 24 h of life (T0), 21 (IQR 15–22) at 24–48 h of life (T1), 14 (IQR 12–18) within 12 h of surgical repair (T2) and 4 (IQR 2–15) a week after the surgical repair (T3). The CDH-LUS significantly dropped over time from the first 24 h of life (T0) to a week after the surgical repair (T3), according to ANOVA for repeated measures. ***Conclusion:*** We showed a significant improvement in CDH-LUS scores from the immediate postoperative period, with normal ultrasonographic evaluations a week after surgery in most patients.

## 1. Introduction

Congenital diaphragmatic hernia (CDH) is a rare congenital malformation affecting 2.5 cases in 10,000 births. The herniation of abdominal viscera into the thorax is associated with varying degrees of pulmonary hypoplasia, cardiac dysfunction, and pulmonary hypertension [1]. Despite advances in therapy, mortality remains high, with rates of up to 30% [2]. Since CDH patients represent a high-risk population, maximal clinical and instrumental monitoring (pulse oximetry, hemodynamic monitoring, near-infrared spectroscopy, cerebral function monitoring, and echocardiography) is recommended to minimize CDH-related complications and improve clinical outcomes [3].

Ultrasonographic (US) features of CDH patients have been widely described during fetal life, with the fetal lung volume used as a surrogate marker for pulmonary hypoplasia, which seems to well predict postnatal outcomes [4]. The lung-area-to-head circumference ratio (LHR) is the most used measurement, comparing the contralateral lung area at the level of a four-chamber heart image to the fetal head circumference. It has been demonstrated that lung size is more accurately expressed as the ratio of the observed LHR of the index case to the expected LHR age-matched controls (observed/expected LHR, O/E LHR) [4]. Based on the O/E LHR, pulmonary hypoplasia in left-sided CDH can be classified as extreme (<15%), severe (15–25%), moderate (26–45%), or mild (46% and higher), with expected postnatal survival of <5%, 20%, 55%, and 85%, respectively. Such classification can currently be used as a guide in prenatal management [5]. The available data about the postnatal use of ultrasonography to assess CDH neonates is scarce. Corsini et al. used postnatal lung ultrasonography (LUS) aiming to evaluate ultrasonographic anatomical findings in patients with CDH and identified a partial absence of the pleural line and therefore the absence of pleural sliding in the affected hemithorax; absence of A-lines in the affected area; a multilayered area with hyperechoic contents in motion, typical of the normal gut and its peristalsis. In the case of right-sided CDH, they also identified the liver in the thorax [6]. The use of an LUS score has been described in the early phases of neonatal respiratory distress syndrome [7]; however, there is still no data about the application of the LUS score to CDH patients. The use of the LUS score as a bedside diagnostic technique in neonates with CDH could help clinicians optimize the ventilatory settings and hemodynamics before and after surgery in a more personalized, dynamic, and noninvasive way compared to traditional chest X-rays.

This study aims to describe the postnatal LUS score patterns in neonates with CDH in order to assess its feasibility and reliability as a diagnostic tool in case of such rare congenital malformation.

## 2. Materials and Methods

### 2.1. Study Design

The present retrospective observational cross-sectional study included all consecutive neonates with a diagnosis of CDH admitted to our Neonatal Intensive Care Unit (NICU) from June 2022 to December 2022 who underwent lung ultrasonography. During the study period, CDH patients were managed according to standard protocols (CDH EURO Consortium Consensus) [8]. Patients’ data (demographics, prenatal data, ventilation strategies, hemodynamics details, and defect type) were retrospectively obtained from electronic medical records.

### 2.2. Lung Ultrasound Examinations

Lung ultrasonography (LUS) was determined at scheduled time points: (T0) during the first 24 h of life; (T1) at 24–48 h of life; (T2) within 12 h of surgical repair; (T3) a week after the surgical repair. To minimize neonatal discomfort, LUS assessments were performed exclusively after the patient’s routine care, during sedation (at T0, T1, and T2), and during quiet spontaneous sleep and/or sedation (at T3). Images were obtained by experienced neonatologists (C.M., D.U.D.R., S.R., I.B., L.M., S.C., and F.C.) using a pocket-sized “iViZ” wireless ultrasound scanner (Fujifilm Sonosite Inc., Bothell, WA, USA) with a linear probe (13 MHz), in agreement with Gomond-Le Goff et al. [9]. B-mode views were used to evaluate the diaphragm during the lung ultrasound examination.

**Figure 1:**
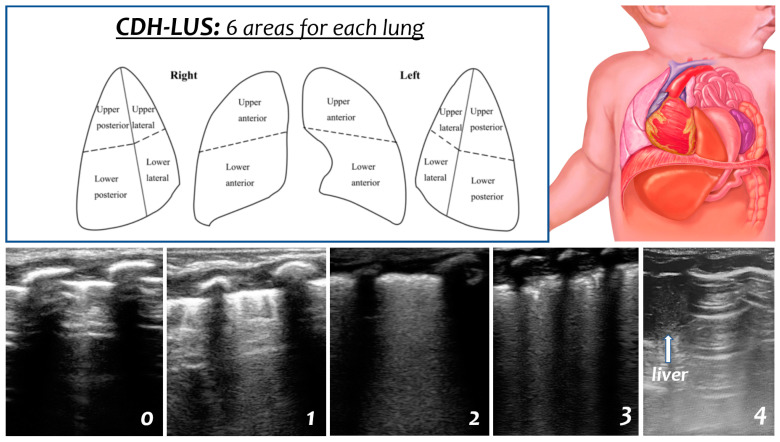
CDH-LUS score description. Each lung is divided into six areas, and a score from 0 to 4 can be given to each scan as follows: 0 indicates an A-pattern (defined by the presence of only A-lines), 1 indicates a B-pattern (defined as the presence of ≥3 B-lines), 2 indicates a severe B-pattern (defined as the presence of crowded and coalescent B-lines with or without consolidations limited to the subpleural space), 3 indicates extended consolidations, and 4 indicates the presence of herniated viscera in the hemithorax (liver, small bowel, stomach, or heart in the case of a mediastinal shift) in the preoperative scans and pleural effusions in the postoperative scans.

We used a modified LUS score, starting from the original 0–3 score by Brat et al. [10]. To assess the modified LUS score for CDH infants (CDH-LUS), we divided each lung into six areas, and a score from 0 to 4 was assigned to each scan as follows: 0 indicates A-pattern (defined by the presence of only A-lines), 1 indicates B-pattern (defined as the presence of ≥3 B-lines), 2 indicates a severe B-pattern (defined as the presence of crowded and coalescent B-lines with or without consolidations limited to the subpleural space), 3 indicates extended consolidations, and 4 indicates the presence of herniated viscera in the hemithorax (liver, small bowel, stomach, or heart in the case of a mediastinal shift) in the preoperative scans and pleural effusions in the postoperative scans (Figure 1). We defined a total CDH-LUS score (ranging from 0 to 48) from 0–12 as mild, 13–24 as moderate, or ≥25 as severe. Clinicians involved in postnatal management were blinded about CDH-LUS results, and scores were retrospectively collected.

### 2.3. Objectives

The primary objective was to describe the global CDH-LUS score trajectory over the NICU admission. The secondary objective was to describe the separate CDH-LUS scores for the lung affected by CDH (ipsilateral CDH-LUS score, iLUS) and the contralateral lung (contralateral CDH-LUS score, cLUS).

### 2.4. Ethical Approval and Statistical Analysis

All procedures performed in this study were in accordance with the ethical standards of the institutional and national research committee and with the 1964 Helsinki Declaration and its later amendments or comparable ethical standards. The study was approved by our Scientific Directorate, and as a retrospective analysis with no patient-identifiable information, it was approved without the need for written consent. Personal data were restricted to essential information and were treated to guarantee the respect of the involved patients’ privacies, as stated by Italian Law D. Lgs. N. 196 of 2003 regarding personal data protection.

Data are presented as numbers and percentages for categorical variables. Continuous variables are expressed as means ± standard deviation if they were normally distributed or as the median and interquartile range (IQR) if normality could not be accepted, according to the D’Agostino–Pearson test. Changes in CDH-LUS scores at different time points were compared with analysis of variance (ANOVA) for repeated measures with a Huynh–Feldt correction (when epsilon is greater than 0.75) or Greenhouse–Geisser correction (preferred when epsilon < 0.75). As appropriate, comparisons between groups were made with a *t*-test or Mann–Whitney test. A *p*-value < 0.05 was considered significant. Statistical analysis was performed using software programs Microsoft Excel (2016 for Windows) and MedCalc (version 12.7 for Windows).

## 3. Results

In this retrospective observational cross-sectional study, we evaluated the medical records of 14 consecutive CDH infants admitted to our NICU during the study period. After excluding a patient who died in the first hours of life and did not undergo lung ultrasonography, 13 neonates with CDH who underwent LUS were included and further analyzed. Demographic and clinical characteristics of the patients are summarized in Table 1. All 13 included patients had a prenatal diagnosis, were inborn, and survived. Twelve/13 had a left-sided hernia (2 severe, 3 moderate, and 7 mild cases), while one patient had a right-sided severe hernia. The mean duration of mechanical ventilation was 5 ± 2 days, whereas the median duration of oxygen requirement was 3 days (IQR 2–10). Surfactant was initially administered only to a single late preterm infant (7.7%). Ten infants (76.9%) required high-frequency oscillatory ventilation (HFOV) and also required a median of a further 4 days (IQR 2–6) of noninvasive ventilation after extubation. Ten patients (76.9%) were treated with milrinone. One patient (7.7%) also required inhaled nitric oxide, and another one (7.7%) intravenous sildenafil because of pulmonary hypertension.

**Figure 2:**
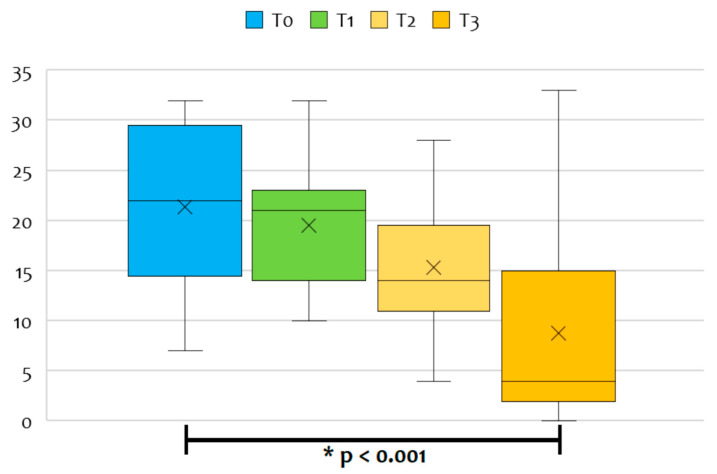
The median CDH-LUS score during the first 24 h of life (T0), at 24–48 h of life (T1), within 12 h of surgical repair (T2), and a week after the surgical repair (T3).

The median CDH-LUS score was 22 (IQR 16–28) during the first 24 h of life (T0), 21 (IQR 15–22) at 24–48 h of life (T1), 14 (IQR 12–18) within 12 h of surgical repair (T2) and 4 (IQR 2–15) a week after the surgical repair (T3) (Figure 2). Five patients (38.5 %) developed a postoperative pleural effusion that was monitored daily: in two cases, a pleural drainage tube was left in after surgery, while in the other three cases, the medical team opted for a wait-and-see approach until spontaneous resolution.

When CDH-LUS scores were analyzed independently for the single fields, 11 patients (84.6%) had scores of 4 at preoperative scans (with the viscera herniation), especially in the inferior fields of the ipsilateral lung. In these patients, a score of 4 was given to a median of three fields per patient (IQR: 3–4; range: 2–7) during the first 24 h of life (T0) and to a median of two fields per patient (IQR: 2–4; range: 1–7) at 24–48 h of life (T1). 

Conversely, only five patients (38.5%)—those who developed pleural effusions—were given a score of 4 at postoperative scans. In the postoperative scans of these five patients, a score of 4 was given to a median of three fields per patient (IQR: 2–4; range: 1–5) within 12 h of surgical repair (T2) and to two fields per patient (IQR: 2–3; range: 0–4) a week after the surgical repair (T3), due to the gradually improved lung aeration.

The CDH-LUS significantly dropped over time from the first 24 h of life (T0) to a week after the surgical repair (T3), according to ANOVA for repeated measures with a Huynh–Feldt correction (*p* < 0.001). Conversely, we found no significant differences in pairwise comparisons between CDH-LUS scores at the first three time points (Table 2).

All patients but one were assisted with invasive mechanical ventilation (MV) at T0 and T1; one patient underwent a thoracoscopic repair within 48 h of life and was extubated immediately after surgery.

At the postoperative evaluation (T2), three patients assisted with noninvasive ventilation (NIV) had mild-to-moderate scores (<18), whereas ten patients assisted with invasive MV presented with higher scores (moderate-to-severe scores, up to 28), but without significant differences (*p* = 0.469).

At 7 days postsurgery evaluation (T3), eight patients who were in spontaneous breathing without the need for respiratory assistance nor oxygen therapy had mild CDH-LUS scores (median score 2.5; IQR 1.5–3.5), while three patients who still needed NIV had mild-to-moderate CDH-LUS scores (median score 15; IQR 12–20) (*p* < 0.001).

Among eleven patients who were successfully extubated before the seventh postoperative day, most had a mild CDH-LUS score corresponding to normally aerated lungs (median score 2; IQR 0.5–3), whereas higher scores were assigned to those patients presenting pleural effusions (median score 11.5; IQR 6–20) (*p* = 0.006). Moreover, the two patients who were still intubated had moderate-to-severe CDH-LUS scores (median score 24; IQR 20–29).

When separately analyzing CDH-LUS scores for the lung affected by CDH (ipsilateral CDH-LUS score, iLUS) and the contralateral lung (contralateral CDH-LUS score, cLUS), we noted that both scores significantly decreased over time from the first 24 h of life (T0) to a week after the surgical repair (T3), according to ANOVA for repeated measures with a Huynh–Feldt correction (*p* < 0.001 for iLUS, and *p* = 0.035 for cLUS, respectively).

However, pairwise comparisons at different time points with a Bonferroni post hoc test showed significant differences only for iLUS values (Table 3), with no significant differences for cLUS values (Table 4).

**Figure 3:**
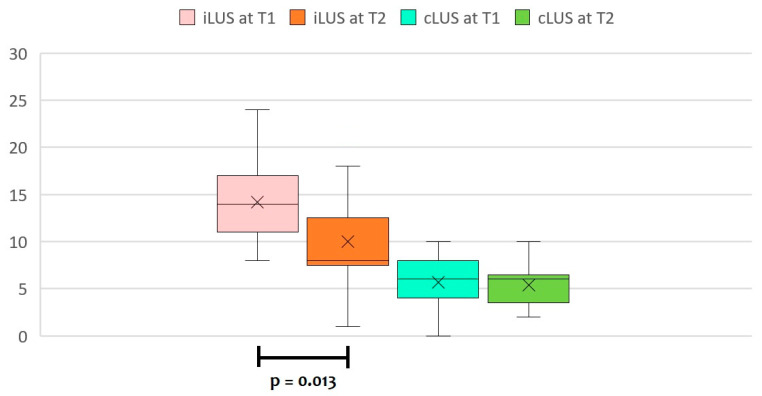
The median CDH-LUS score for the lung affected by CDH (ipsilateral CDH-LUS score, iLUS) and for the contralateral lung (contralateral CDH-LUS score, cLUS) at T1 (24–48 h of life) and T2 (within 12 h of surgical repair).

We already found significant differences in iLUS values between T1 and T2 (Figure 3)**.**

## 4. Discussion

To our knowledge, this is the first study describing the trajectory of LUS scores in neonates affected by CDH.

Lung ultrasound has become more and more popular in the last decade, both in neonatal and pediatric age groups. Several studies addressed the usefulness of this procedure mainly because of its possibility to be utilized at the bedside, reducing the exposure to ionizing radiation along with a simple and immediate interpretation of the images [11,12]. Specific LUS patterns can help diagnose most infant lung disorders; such imaging techniques are also being employed as a semiquantitative method or, more recently, as a “functional” tool [13]. LUS score describes lung parenchyma aeration and encompasses the total spectrum of possible pulmonary conditions (a normally aerated lung, an interstitial pattern, an alveolar pattern, or consolidations) [10]. Indeed, several studies have shown the reliability of the LUS score in predicting the need for surfactant replacement therapy and/or noninvasive and mechanical ventilation in preterm infants [7,14,15,16,17]. Recently, Raimondi et al. demonstrated that in preterm neonates affected by respiratory distress syndrome, the LUS trajectory is gestational age-dependent, significantly correlates with the oxygenation status, and predicts bronchopulmonary dysplasia [7].

In congenital diaphragmatic hernia (CDH), the specific anatomy of the diaphragm defect, the contribution of additional congenital anomalies, the degree of lung hypoplasia and pulmonary hypertension, as well as overall cardiopulmonary function, lead to a highly varied clinical presentation: risk stratification is thus crucial to guide postnatal management [18].

We demonstrated a trend of improvement in CDH-LUS scores over the postoperative period, with normal ultrasonographic evaluations a week after surgery in most survivors. In particular, iLUS scores significantly decreased after surgical repair, rather than cLUS.

Our data show the potentiality of LUS-based scores to monitor changes in lung volumes in neonates with CDH before and after surgical repair. We propose that the timing of CDH-LUS evaluation could be: (1) at birth, (2) before surgical repair, (3) after surgical repair, and (4) at weaning from mechanical ventilation/extubation. A possible use of this score could be the identification of the earlier moment when to wean these infants from mechanical ventilation, reducing the duration of mechanical ventilation and related side effects, but should be prospectively evaluated.

Our CDH-LUS score could open a research field and help clinicians in the optimization of ventilatory settings and hemodynamics, along with targeted echocardiography [19]. Indeed, point-of-care ultrasound (POCUS) is an emerging clinical tool in constant growth in neonatal intensive care: current evidence supports the use of POCUS for several diagnostic and procedural applications in a noninvasive way [20,21]. Surprisingly, this enthusiasm has yet to involve the management of CDH, probably due to the rarity of this disease and the greater ease of enrolling preterm infants in clinical studies about lung ultrasonography.

The originality of this study resides mostly in the modified LUS score (4 points per field rather than 3) in the presence of herniated viscera in the ipsilateral hemithorax (liver, small bowel, stomach, or heart in the case of a mediastinal shift) in the preoperative scans and pleural effusions in the postoperative scans. In the preoperative scans, we assigned a score of 4 to most patients (84.6%), whereas, in the postoperative scans, less than half (38.5%) had a score of 4 in some fields. Indeed, surgical repair, bringing the viscera back into the abdomen, allowed a reorganization of the space inside the affected hemithorax and a gradual re-expansion of the ipsilateral lung, demonstrated by lower scores at postoperative LUS evaluation.

Interestingly, we noted that the number of lung fields to whom a score of 4 has been assigned was lower during the second 24 h of life rather than the first 24 h of life, demonstrating that mechanical ventilation has a crucial role in recruiting all lung areas before elective surgical repair.

Our retrospective observational cross-sectional study has some limitations: it was a single-center study, and it was conducted on a small sample and short time, although we should consider that CDH is a rare disease. Moreover, we are not able to report data about LUS scores in patients who did not survive. Further investigations are needed in a larger multicenter sample and in a prospective way to confirm our results and better define the CDH-LUS score’s role in the postnatal management of CDH infants, reducing their exposure to ionizing radiation. Indeed, our next aim will be to prospectively study the correlation between CDH-LUS scores and clinical prenatal and postnatal variables, such as the observed/expected LHR (O/E LHR), the duration of mechanical ventilation (MV), the need for high-frequency oscillatory ventilation (HFOV), the duration of oxygen requirement, the length of hospital stays, and survival.

Lastly, growing attention should be paid to lung volumes of infants with CDH, given that those with hypoplasic lungs have a significantly lower weight at 12 months and at 24 months when compared to infants with normal lungs [22]. Infants with CDH often have an initial growth restriction with loss-of-weight Z-scores during the NICU stay, as we evidenced in our small cohort. Considering that patients with hypoplastic lungs should receive more aggressive nutritional support, the correlation between CDH-LUS score and separate lung volume measurements should also be studied in a larger sample in the future to provide an easy predictor of increased caloric needs in centers where specific lung volume measurements are not available.

## 5. Conclusions

We confirm that a modified LUS score can also be used in CDH infants, with appropriate changes due to the presence of herniated viscera in the ipsilateral hemithorax before surgical repair and the presence of pleural effusion in the postoperative period. We showed a significant improvement in CDH-LUS scores from the immediate postoperative period, with normal ultrasonographic evaluations a week after surgery in most patients. This novel score could be used to evaluate bedside changes in lung aeration during the initial clinical stabilization to decide the best moment to perform the elective surgical repair. Furthermore, it could be used to assess changes in lung fields after surgical repair, helping clinicians to objectively decide when infants are ready to be weaned from mechanical ventilation. Therefore, further prospective multicenter studies are needed to explore all of the potential roles of LUS in CDH patients.

## Figures and Tables

**Table 1:** Characteristics of CDH infants included in the study. Results are expressed as numbers (percentages) and means (± standard deviation) or medians (IQR).

Characteristics	CDH Patients (*n* = 13)
Males	7 (53.8%)
Gestational age (weeks)	37 (36–38)
Late preterm (34–36 weeks)	4 (30.8%)
Birthweight (grams)	2878 ± 682
Birthweight Z-score (SDS)	0.06 (−0.89/0.39)
Small for gestational age at birth	1 (7.7%)
Observed/expected lung-area-to-head circumference ratio	41 ± 12
Fetal endoluminal tracheal occlusion (FETO)	4 (30.8%)
Liver up	6 (46.2%)
Defect size	
- A	2 (15.3%)
- B	5 (38.5%)
- C	5 (38.5%)
- D	1 (7.7%)
Patch repair	4 (30.8%)
Age at surgery (hours of life)	77 ± 43
Length of hospital stay (days)	33 ± 21
Weight at discharge (grams)	3306 ± 539
Infants with a loss-of-weight Z-score of >1 SDS from birth to discharge	10 (76.9%)

**Table 2:** Pairwise comparisons between CDH-LUS scores at different time points with a Bonferroni post hoc test.

Factors			Mean Difference	Std. Error	95% CI	*p*-Value
LUS_T0	-	LUS_T1	1.846	1.480	−2.820 to 6.512	1.000
	-	LUS_T2	6.077	2.376	−1.415 to 13.569	0.151
	-	LUS_T3	12.538	2.958	3.212 to 21.865	0.007
LUS_T1	-	LUS_T0	−1.846	1.480	−6.512 to 2.820	1.000
	-	LUS_T2	4.231	1.791	−1.415 to 9.877	0.215
	-	LUS_T3	10.692	2.540	2.683 to 18.701	0.007
LUS_T2	-	LUS_T0	−6.077	2.376	−13.569 to 1.415	0.151
	-	LUS_T1	−4.231	1.791	−9.877 to 1.415	0.215
	-	LUS_T3	6.462	1.920	0.408 to 12.515	0.034
LUS_T3	-	LUS_T0	−12.538	2.958	−21.865 to −3.212	0.007
	-	LUS_T1	−10.692	2.540	−18.701 to −2.683	0.007
	-	LUS_T2	−6.462	1.920	−12.515 to −0.408	0.034

**Table 3:** Pairwise comparisons between ipsilateral CDH-LUS (iLUS) scores at different time points with a Bonferroni post hoc test.

Factors			Mean Difference	Std. Error	95% CI	*p*-Value
iLUS_T0	-	iLUS_T1	0.538	0.931	−2.397 to 3.474	1.000
	-	iLUS_T2	6.154	1.609	1.082 to 11.225	0.015
	-	iLUS_T3	8.692	1.889	2.736 to 14.648	0.004
iLUS_T1	-	iLUS_T0	−0.538	0.931	−3.474 to 2.397	1.000
	-	iLUS_T2	5.615	1.439	1.078 to 10.152	0.013
	-	iLUS_T3	8.154	1.961	1.973 to 14.335	0.008
iLUS_T2	-	iLUS_T0	−6.154	1.609	−11.225 to −1.082	0.015
	-	iLUS_T1	−5.615	1.439	−10.152 to −1.078	0.013
	-	iLUS_T3	2.538	1.505	−2.206 to 7.282	0.705
iLUS_T3	-	iLUS_T0	−8.692	1.889	−14.648 to −2.736	0.004
	-	iLUS_T1	−8.154	1.961	−14.335 to −1.973	0.008
	-	iLUS_T2	−2.538	1.505	−7.282 to 2.206	0.705

**Table 4:** Pairwise comparisons between contralateral CDH-LUS (cLUS) scores at different time points with a Bonferroni post hoc test.

Factors			Mean Difference	Std. Error	95% CI	*p*-Value
cLUS_T0	-	cLUS_T1	1.308	0.873	−1.443 to 4.059	0.959
	-	cLUS_T2	−0.077	1.693	−5.413 to 5.259	1.000
	-	cLUS_T3	3.923	1.398	−0.485 to 8.331	0.095
cLUS_T1	-	cLUS_T0	−1.308	0.873	−4.059 to 1.443	0.959
	-	cLUS_T2	−1.385	1.734	−6.851 to 4.082	1.000
	-	cLUS_T3	2.615	1.059	−0.725 to 5.955	0.177
cLUS_T2	-	cLUS_T0	0.077	1.693	−5.259 to 5.413	1.000
	-	cLUS_T1	1.385	1.734	−4.082 to 6.851	1.000
	-	cLUS_T3	4.000	1.605	−1.061 to 9.061	0.170
cLUS_T3	-	cLUS_T0	−3.923	1.398	−8.331 to 0.485	0.095
	-	cLUS_T1	−2.615	1.059	−5.955 to 0.725	0.177
	-	cLUS_T2	−4.000	1.605	−9.061 to 1.061	0.170

## Data Availability

All data considered in this study are reported in this article.
